# A Systematic Review and Meta-analysis of Prophylactic versus Pre-emptive Strategies for Preventing Cytomegalovirus Infection in Renal Transplant Recipients

**Published:** 2012-02-01

**Authors:** B. B. Rawal, S. Shadrou, F. Abubacker, N. Ghahramani

**Affiliations:** Pennsylvania State University College of Medicine, Hershey Medical Center, Department of Medicine, Division of Nephrology, USA

**Keywords:** Cytomegalovirus, Prophylactic, Pre-emptive, Kidney transplantation, Rejection

## Abstract

Background: In kidney transplant (KT) recipients, CMV infection poses significant morbidity and mortality. Both prophylactic and pre-emptive approaches for preventing CMV infection have been utilized.

**Objective**: To compare the effectiveness of routine prophylaxis *vs.* pre-emptive treatment for preventing CMV disease after KT.

**Methods**: We conducted a systematic review and meta-analysis comparing the effectiveness of routine prophylaxis *vs.* pre-emptive treatment for preventing CMV disease after KT. Combining 4 comprehensive search terms (CMV, renal transplant, prophylaxis, pre-emptive); we searched PubMed, EMBASE, ISI Web of Science, and Cochrane Central Register from inception through January 2011. We also evaluated studies referenced in review articles and abstracts from meetings of major nephrology and transplant societies (2009–2011). Two authors independently extracted data and assessed methodological criteria. The primary outcome was the pooled estimate of the odds ratio (OR) of developing CMV infection. Secondary outcomes included OR of acute rejection, OR of graft loss and OR of death within first year of KT. Comprehensive Meta-analysis V2 software was used for data analysis.

**Results**: Analysis of 9 randomized controlled trials (991 patients; ganciclovir=5, valganciclovir=4) with CMV infection as an outcome revealed the OR of CMV infection to be 0.34 (95% CI: 0.25–0.46, p=0.008) for the prophylactic *vs.* the pre-emptive groups. The OR of acute rejection (7 studies; 1358 patients) was 0.52 (95% CI: 0.41–0.67, p=0.001) with prophylactic approach compared to pre-emptive treatment; graft loss (7 studies; OR 0.52 [95% CI: 0.34–1.12, p=0.32] and mortality (6 studies; OR 0.84 [95% CI: 0.62–1.23, p=0.23]) were similar between the two groups.

**Conclusions**: Prophylactic approach is superior to pre-emptive approach in preventing CMV infection within the first year of kidney transplant. The risk of developing acute rejection is also lower with prophylactic approach in the first year of transplant but there is no significant difference in graft loss or mortality with either approach.

## INTRODUCTION

In organ transplant recipients, the risk of opportunistic infection is significantly higher than in the general population as a result of exposure to chronic immunosuppression. Cytomegalovirus (CMV) remains a leading cause of such opportunistic infection within the first year of kidney transplantation [1], and is a major contributor to morbidity and mortality [2]. Latent CMV infection can be present in 60%–90% of all kidney transplant patients. Without routine preventative therapy with anti-viral agents, symptomatic CMV infection occurs in about 20%–60% of cases, mostly within the first three months after transplantation [3,4]. Late-onset CMV disease, occurring between 3–6 months after kidney transplant, is also frequently encountered in approximately 30%–40% of high risk patients. The risk of CMV infection is highest in CMV seronegative recipients (R) receiving kidney from CMV seropositive donors (D+) (D+/R status) [5-7] and in patients who are exposed to lymphocyte-depleting induction agents such as thymoglobulin [6,8]. CMV infection can manifest in the form of asymptomatic CMV viremia or mild viral syndrome to severe tissue-invasive disease (*e.g.*, pneumonitis, hepatitis, colitis, esophagitis, encephalitis, *etc*). CMV infection has also been associated with increased risk of acute graft rejection [9,10] which is known to adversely impact long-term graft survival [11]. The mortality rate from untreated, tissue-invasive CMV disease is more than 60% [12]. Needless to say, the overall cost associated with the disease burden, including hospitalization, diagnostic testing (such as CMV PCR) and treatment with anti-viral drugs is overwhelming. Several randomized controlled trials have shown proven benefits of using anti-viral agents—acyclovir, valacyclovir, oral ganciclovir, and valganciclovir—in successfully preventing CMV infection in renal transplant recipients [6,13-15]. Currently, two standard approaches have been considered acceptable for preventing CMV infection after kidney transplantation: universal prophylaxis approach and pre-emptive therapy. Depending on the experience or preference of a transplant center, either of the two approaches is utilized for CMV disease prevention. There are numerous retrospective studies and prospective, randomized trials [16-24] that have compared the effectiveness of universal prophylaxis with pre-emptive therapy in CMV disease prevention after kidney transplant. However, the conflicting results of these studies have led to confusion regarding superiority of one approach to another. The objective of our study was to compare the two commonly practiced approaches by a systematic review and meta-analysis, and to also evaluate the risks of acute graft rejection, graft loss and mortality within one year of kidney transplant.

## METHODS

Literature Search 

Four comprehensive search terms were combined for the literature search: “CMV,” “renal transplant,” “prophylaxis,” and “pre-emptive.” An extensive database search was done from PubMed, EMBASE, ISI Web of Science, and Cochrane Central Register from their inception through January 2011. Studies referenced in review articles and abstracts from meetings of major nephrology and transplant societies (between 2009 and 2011) were also included. 

*Study Criteria*

Inclusion criteria

All randomized controlled trials (RCTs) that aimed at comparing universal prophylaxis with pre-emptive approach for preventing CMV infection after kidney transplant irrespective of: 1) the type of anti-viral agent (*e.g*., valacyclovir *vs*. valganciclovir); 2) the dose of anti-viral agent (*e.g*., valganciclovir 450 mg *vs*. 900 mg daily); 3) the duration of prophylaxis (3 months *vs*. 6 months); and 4) the language of publication. 

Exclusion criteria

1) studies with insufficient data; 2) duplicate studies; 3) single case reports; 4) review articles; and 5) studies of CMV in organ transplants other than kidney.

Two of the authors (BR and NG) independently evaluated articles for eligibility in a two-stage procedure. In the first stage, the abstracts of all identified studies were reviewed. The second stage consisted of full-text review of studies that met the inclusion criteria or those for which eligibility was uncertain. Articles that were selected by either individual were reviewed by both authors and evaluated on both inclusion and exclusion criteria. Disagreement between authors was resolved by consensus. 

Data Extraction, Definitions and Outcomes 

Data extracted included study design, authors, publication year, number of patient (n), details of treatment, and data on the following outcomes: development of CMV infection, graft loss and death within the first year. The primary outcome was the pooled estimate of the odds ratios (OR) of developing CMV infection within one year of transplant. Secondary outcomes included odds ratios of acute rejection, graft loss, and death within one year of transplant. The following definitions were used during data extraction and analysis:

CMV infection: Asymptomatic CMV viremia or tissue-invasive CMV disease.

CMV viremia: Elevated CMV DNA in recipient’s blood quantified by CMV PCR.

CMV disease: CMV syndrome (fever >38 °C, fatigue, leukopenia) with CMV viremia as well as organ involvement by clinical or histopathological findings (pneumonitis, hepatitis, colitis, esophagitis, *etc*).

Acute rejection: Acute allograft rejection reported on graft biopsy within 12 months after transplant.

Graft loss: Allograft failure requiring dialysis or repeat transplant within 12 months.

Mortality: All-cause mortality reported within 12 months after transplant.

Universal Prophylaxis: All at-risk kidney transplant recipients receive anti-viral agent for at least three months after their transplant.

Pre-emptive therapy: Anti-viral agent is not routinely given to kidney transplant recipients. Instead, their CMV PCR is monitored at frequent intervals to detect early evidence of CMV replication. If CMV PCR is found to be elevated beyond a set cut-off, such patient receives a treatment dose of anti-viral agent regardless of symptoms.

Statistical Analysis 

We used the Mantel-Haenszel model to estimate the pooled OR with 95% confidence intervals (95% CI) for study outcomes under the fixed effect model, using data from all eligible RCTs. The presence of heterogeneity across trials was evaluated using Q-statistic for heterogeneity. The heterogeneity statistic was then incorporated to calculate the summary OR under the random effects model [25]. All statistical analyses were performed using Comprehensive Meta-Analysis ver 2.2.057 (Englewood, NJ).

## RESULTS

**Figure 1:**
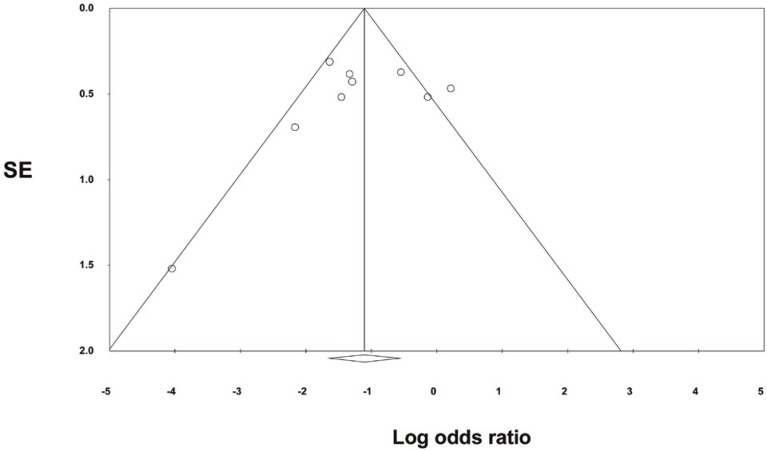
Funnel plot of standard error (SE) by Log Odds Ratio

**Figure 2:**
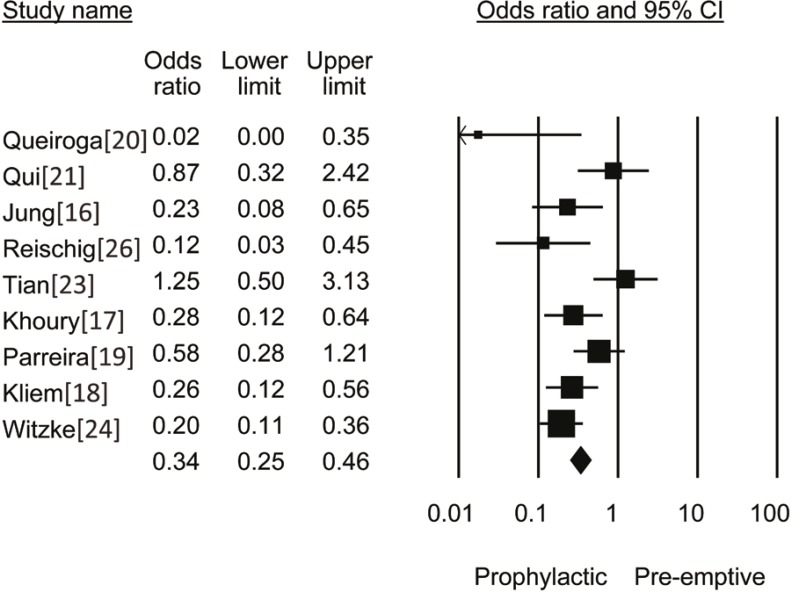
OR of CMV Infection with Prophylactic *vs*. Pre-emptive Approach

**Figure 3:**
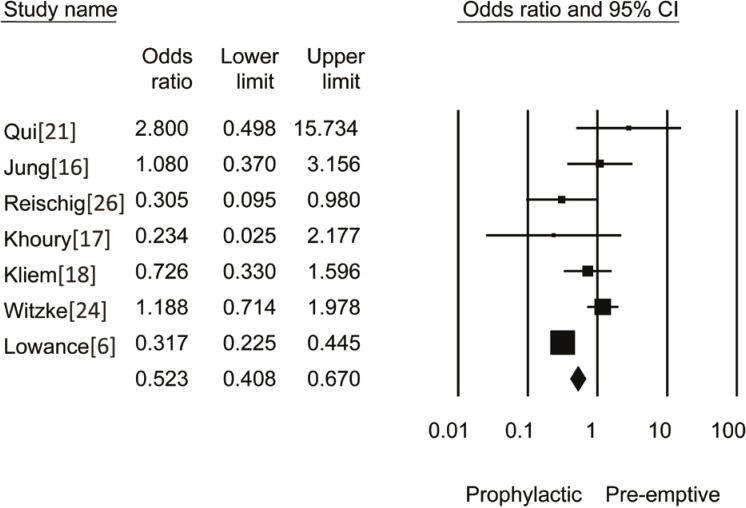
OR of acute rejection with prophylactic *vs.* pre-emptive approach

Our search resulted in 242 reports. Nine randomized controlled trials (991 patients) reported on CMV infection within one year of transplantation; six of these studies, as well as one additional study [6], reported on acute rejection within the first year (1358 patients) (Table 1). The funnel plot of the trials is shown in Figure 1. Analysis of the nine RCTs (five studies with ganciclovir and four with valganciclovir) revealed that the OR of CMV infection with prophylaxis *vs*. pre-emptive therapy was 0.34 (95% CI: 0.25–0.46, p=0.008) (Fig 2). Analysis of seven RCTs (n=1358) with data on acute rejection at one year, showed that the OR of acute rejection with prophylaxis *vs*. pre-emptive therapy was 0.52 (95% CI: 0.41–0.67, p=0.001) (Fig 3). OR of graft loss within one year of transplant with prophylactic approach as compared to pre-emptive therapy was 0.52 (95% CI: 0.34–1.12, p=0.32). OR of death within one year of transplant with prophylactic approach as compared to pre-emptive therapy was 0.84 (95% CI: 0.62–1.23, p=0.23). 

**Table 1:** Study characteristics

Study	Total	Prophylactic agent (n)	Pre-emptive agent (n)	Outcomes	Mean follow-up (months)
Queiroga (2003)[20]	34	Ganciclovir (9)	Ganciclovir (25)	CMVI*, CMVD**, graft loss, mortality, cost	6
Qui (2008)[21]	60	Ganciclovir (30)	Ganciclovir (30)	CMVI, CMVD, rejection, graft loss	6
Jung (2001)[16]	70	Ganciclovir (34)	Ganciclovir (36)	CMVI, CMVD, rejection, graft loss, cost	12
Reischig (2008)[27]	70	Valacyclovir (34)	Valganciclovir (36)	CMVI, CMVD, rejection, costs	12
Tian (2005)[23]	80	Ganciclovir (40)	Ganciclovir (40)	CMVI, CMVD	12
Khoury (2006)[17]	98	Valganciclovir (49)	Valganciclovir (49)	CMVI, CMVD, rejection, cost	12
Parreira (2009)[19]	135	Valganciclovir (51)	Valganciclovir (84)	CMVI, CMVD	12
Kliem (2008)[18]	148	Ganciclovir (74)	Ganciclovir (74)	CMVI, rejection, graft function, mortality	48
Witzke (2011)[24]	296	Valganciclovir(146)	Valganciclovir 150	CMVI, CMVD, rejection, graft function	12
Lowance (1999)[6]	616	Valacyclovir (306)	Placebo (310)	CMVD, rejection	6
Total	1607	773	834	–	–

* CMVI: Cytomegalovirus infection

** CMVD: Cytomegalovirus disease

## DISCUSSION

We found that prophylactic approach is superior to pre-emptive treatment in preventing CMV infection within the first year of kidney transplant. We also found that the risk of developing acute graft rejection with universal prophylaxis is lower, but there is no significant difference in the risk of graft loss or mortality in the first year with either approach. 

With prophylactic approach, there is always a potential concern for development of late-onset CMV disease (occurring >3 months) since the duration of prophylaxis is typically not more than three months. Although mixed results exist, double blind RCTs favor six months prophylaxis for preventing late-onset CMV infection [26]. In our review, we found that the overall risk of developing late CMV infection within 12 months with prophylactic approach as compared to pre-emptive treatment is still lower, regardless of three or six months of prophylaxis. In addition, our study showed less risk of graft rejection with prophylactic approach. Out of seven RCTs (n=1358) that were reviewed to assess risk of acute rejection, six were small-sized studies that showed no significant difference in risk of acute rejection with either approach. The only large study [6] showed definitive reduction in the risk of acute rejection with prophylactic approach (OR=0.32, 95% CI: 0.23–0.45). Since this study included 616 participants (45% of total patients in seven RCTs), the pooled estimate of the OR of rejection was shifted in favor of universal prophylaxis. More RCTs with larger sample size are warranted to validate this finding.

Pre-emptive therapy does have advantages of its own. This approach requires frequent monitoring of CMV replication (by checking CMV DNA PCR in blood), and CMV infection is less likely to be missed or go untreated if asymptomatic viral replication is detected early. However, the exact cut-off of elevated CMV PCR for initiating treatment with anti-viral drugs has not been well established. Different transplant centers use variable cut-offs ranging from 1000 to 2000 copies of CMV PCR. This can lead to overtreatment in patients (if cut-off used is too low) who may otherwise never have developed symptomatic CMV disease. As a result, they may be exposed to unnecessary side-effects of such anti-viral agents and increased cost associated with the therapy. On the other hand, this can also lead to under-treatment (if cut-off used is too high) from failure to detect asymptomatic viremia that may eventually progress to develop symptomatic disease. Asymptomatic CMV viremia has been shown to reduce graft survival and increase mortality (hazard ratio of 2.9) [12].

Using prophylactic approach has some limitations too. The risk of developing symptomatic CMV disease in high-risk groups with D+/R serology without anti-viral prophylaxis is about 60% [22]. Therefore, almost 40% of such patients receive unnecessary prophylaxis that can be quite expensive and cause unwanted side effects. However, the risk of developing clinically significant CMV disease is so high that it justifies use of routine prophylaxis in such high-risk group patients. Cost-effectiveness can be another setback for prophylactic approach. Historically, anti-viral drugs such as acyclovir, valacyclovir and oral ganciclovir have been used for CMV prophylaxis. However, the current trend shows valganciclovir as the preferred agent by most transplant centers. Since valganciclovir is the most expensive drug of all four, the economic benefit of the prophylactic approach has been questioned [17]. In a cost analysis by Reischig and colleagues [27], the average CMV-associated costs per patient were US$ 5525 and US$ 2629 for pre-emptive therapy with valganciclovir and prophylactic valacyclovir, respectively (p < 0.001). However, assuming the cost of US$ 60 per PCR test, there was no difference in overall costs.

Our review has the following limitations: 1) we did not perform a complete cost-effectiveness analysis between the two approaches; 2) we did not compare the risk of CMV infection in populations that received lymphocyte-depleting agents as opposed to other induction agents (as exposure to lymphocyte-depleting agents is considered to be a known risk factor for development of CMV disease); 3) the outcome data is only for one year; and 4) publication bias, a limitation inherent to most meta-analyses, since negative studies are less likely to be reported. 

We concluded that prophylaxis is superior to the pre-emptive approach in preventing CMV infection within the first year of kidney transplant and that the risk of developing acute rejection is also lower with prophylaxis than the pre-emptive approach within the first year. Although prophylactic approach favored less risk of acute rejection, there was no difference in graft loss or mortality in one year. However, acute rejection can lead to graft loss earlier than in patients with no rejection. Long-term follow-up data are warranted to evaluate the risk of graft loss and mortality with both prophylactic and pre-emptive approaches. While the two approaches might result in similar costs, since a major driver of the cost in the pre-emptive approach is the cost of CMV PCR testing, if individual transplant centers have the capability of performing the test, the pre-emptive approach might prove to be more cost-effective. We found no significant difference in the risk of graft loss or death in the first year of kidney tranplant with either approach.
